# Neuroimaging in bulimia nervosa and binge eating disorder: a systematic review

**DOI:** 10.1186/s40337-018-0187-1

**Published:** 2018-02-20

**Authors:** Brooke Donnelly, Stephen Touyz, Phillipa Hay, Amy Burton, Janice Russell, Ian Caterson

**Affiliations:** 1 School of Psychology, Clinical Psychology Unit, University of Sydney, Sydney,, New South Wales Australia; 2 Translational Health Research Institute (THRI), School of Medicine, Western Sydney University, Sydney, New South Wales Australia; 3 The Peter Beumont Eating Disorder Service, Royal Prince Alfred Hospital, Sydney, New South Wales Australia; 4 The Boden Institute of Obesity, Nutrition, Exercise and Eating Disorders, University of Sydney, Sydney, New South Wales Australia

**Keywords:** binge eating, binge episode, bulimia nervosa, binge eating disorder, eating disorders, neuroimaging, neurobiology, fMRI

## Abstract

**Objective:**

In recent decades there has been growing interest in the use of neuroimaging techniques to explore the structural and functional brain changes that take place in those with eating disorders. However, to date, the majority of research has focused on patients with anorexia nervosa. This systematic review addresses a gap in the literature by providing an examination of the published literature on the neurobiology of individuals who binge eat; specifically, individuals with bulimia nervosa (BN) and binge eating disorder (BED).

**Methods:**

A systematic review was conducted in accordance with PRISMA guidelines using PubMed, PsycInfo, Medline and Web of Science, and additional hand searches through reference lists. 1,003 papers were identified in the database search. Published studies were included if they were an original research paper written in English; studied humans only; used samples of participants with a diagnosed eating disorder characterised by recurrent binge eating; included a healthy control sample; and reported group comparisons between clinical groups and healthy control groups.

**Results:**

Thirty-two papers were included in the systematic review. Significant heterogeneity in the methods used in the included papers coupled with small sample sizes impeded the interpretation of results. Twenty-one papers utilised functional Magnetic Resonance Imaging (fMRI); seven papers utilized Magnetic Resonance Imaging (MRI) with one of these using both MRI and Positron Emission Technology (PET); three studies used Single-Photon Emission Computed Tomography (SPECT) and one study used PET only. A small number of consistent findings emerged in individuals in the acute phase of illness with BN or BED including: volume reduction and increases across a range of areas; hypoactivity in the frontostriatal circuits; and aberrant responses in the insula, amygdala, middle frontal gyrus and occipital cortex to a range of different stimuli or tasks; a link between illness severity in BN and neural changes; diminished attentional capacity and early learning; and in SPECT studies, increased rCBF in relation to disorder-related stimuli.

**Conclusions:**

Studies included in this review are heterogenous, preventing many robust conclusions from being drawn. The precise neurobiology of BN and BED remains unclear and ongoing, large-scale investigations are required. One clear finding is that illness severity, exclusively defined as the frequency of binge eating or bulimic episodes, is related to greater neural changes. The results of this review indicate additional research is required, particularly extending findings of reduced cortical volumes and diminished activity in regions associated with self-regulation (frontostriatal circuits) and further exploring responses to disorder-related stimuli in people with BN and BED.

## Plain English summary

This paper is a systematic review of research investigating structural and functional differences transdiagnostically; that is, in people who have an eating disorder characterized by binge eating, either Bulimia Nervosa (BN) or Binge Eating Disorder (BED), when compared to healthy people with no eating disorder or other mental illness. Using a set of fixed search terms, we completed a systematic review of published peer-reviewed scientific papers, identifying thirty-two papers that met the inclusion criteria. The majority of papers reviewed used functional Magnetic Resonance Imaging (fMRI) and the rest used one or two other neuroimaging tests. An overview and synthesis of the results of the papers is provided, grouped according to the type of test completed. A small number of findings emerged of individuals with BN or BED when they have clinically significant symptoms, highlighting there are reductions in the overall size of the brain in BN and BED and diminished activity in regions associated with self regulation (frontostriatal circuits). Also, some studies highlight differences in the activity within neural regions associated with emotional processing (amygdala), attention and spatial manipulation (middle frontal gyrus) and visual processing (occipital cortex). We discuss the implications of the results and highlight recommendations for future neurobiological research based on our findings.

### Rationale

Recurrent binge eating is a debilitating symptom that is a core diagnostic criterion for bulimia nervosa (BN) and binge eating disorder (BED); it also occurs in anorexia nervosa-binge purge type (AN-BP), and is a common feature in other specified feeding and eating disorder (OSFED). The Diagnostic and Statistical Manual of Mental Disorders (DSM-5) [1] specifies that individuals are engaging in objective binge episodes (OBEs) at least once per week to reach a diagnosis of BN or BED. This differs from the proposed ICD-11 criterion where the binge episode (BE) is not required to be objectively large and can look like subjective binge episodes (SBEs) [2]. BN involves binge episodes (BEs)^1^ followed by inappropriate compensatory behaviours to avoid weight gain, such as purging, while BED involves engaging in recurrent binge episodes with no compensatory strategies [1].

BN and BED are disorders with noted social and health consequences that typically arise in later adolescent and young adult years [3]. In a cross-sectional population survey of Australian adults, the three-month prevalence of BN and BED ranged from 1.1-1.5% [4]. In 2014 in the Australian population, the prevalence of recurrent binge eating with or without distress was 10.1% and 13.0% in 2015 [3]. Psychiatric comorbidity, particularly with depression and anxiety disorders, is common [5, 7] and mortality is increased [6]. Over one in five individuals with BN will attempt suicide during their life, with factors relating to emotion dysregulation, lifetime anxiety and depression [7].

Treatments and assessments for BN and BED have been developed based on the current definition of BEs. Cognitive Behavioural Therapy (CBT) is the first-line treatment for BN and BED [8]. Available psychological treatments are moderately effective and medication may offer benefits but longer-term maintenance of effects are unclear [9–12]. Research has demonstrated that the majority of individuals with BN and BED do not seek treatment for their eating disorder, but instead present for weight loss treatment [13, 14].

In recent decades, major advances have taken place in the field of neuroscience, which has increased knowledge of the interrelationship between neurological processes and eating disorders [15]. However, most neuroimaging studies have focused their attention on people with AN rather than BN or BED. Presumably, this is because the early neurobiological literature, which focused largely on examining structural changes associated with prolonged starvation and malnutrition in AN with Magnetic Resonance Imaging (MRI), formed a foundation for ongoing investigation in this clinical group.

The neurobiological basis of BN and BED is different to that of AN. Specifically, BN and BED are conceptualized as impulsive / compulsive eating disorders with altered reward sensitivity and food-related attentional biases [16]. Alterations in the cortico-striatal circuits of individuals with BN and BED are similar to those reported in studies of people with substance abuse, with changes in the function of the prefrontal, insular cortex, orbitofrontal cortex (OFC) and striatum [16]. In BED, individuals move from the ventral-striatal reward-based mode of reward-related food consumption, to a dorsal-striatal impulsive / compulsive mode of reward-related food consumption [16]. In BN the urge to binge eat is mediated by hyperactivity of the OFC and anterior cingulate cortex (ACC) and impaired inhibitory control from the lateral prefrontal circuits [17]. Hyperactivity of the parieto-occipital regions and hypoactivation of executive control networks in individuals with BN compared to HCs has also been reported [18].

Increased attention has been directed to the role of inhibitory control, or how well one can suppress inappropriate and unwanted actions, in BN and BED e.g. [18–20]. A recent meta-analysis found impaired response inhibition in BN patients when faced with eating disorder-related stimuli, alongside general impairments in inhibitory control, when compared to HCs [19]. In a recent systematic review, no definite conclusions could be drawn regarding the neurocognitive profile of individuals with BN or BED due to the diversity in methodology and small sample sizes within the majority of the studies reviewed [21]. A smaller body of research has been published over recent years examining the neurobiology of patients who binge eat e.g. [22–24]. The frontostriatal area, which has a central role in controlling goal-directed thoughts and behaviours including response inhibition and reward processing [25], has emerged as particularly relevant to BN [25]. Evidence suggests the diminished frontostriatal brain activation in BN patients contributes to the severity of symptoms [26]. Furthermore, individuals with BN display altered temporal choice behavior, the degree of preference for immediate rewards over delayed rewards [27].

Overall, it is clear that there is a rapidly expanding body of neurobiological research in BN and BED. Completing a rigorous review will provide a novel and warranted overview of the deficits and differences that occur in these clinical groups. This will increase our understanding of the neurological underpinnings of BN and BED, which is critical considering the extremely high rates of psychiatric comorbidities and risk of suicide in people with BN and BED [7, 28]. The pathophysiology of BN and BED is poorly understood and as a result, effective evidence-based treatments require further refinement [27]. Increased knowledge of these factors for BN and BED will better inform treatments across bulimic eating disorders.

### Objective

There has been a recent shift in the treatment framework for eating disorders, to include knowledge of the structural and functional changes in the brain that take place in the ill state. Recent developments in neuroimaging have allowed some clinical and psychopathological symptoms to be linked to specific neural structures and systems. To date, the majority of this research in people with eating disorders has examined individuals with AN. The first aim of this systematic review is to contribute a comprehensive understanding of the existing neuroimaging research in individuals with BN or BED where BEs form the core eating disorder pathology, rather than a possible clinical feature. The second aim relates to the clinical utility of the DSM [1] distinction between OBEs and SBEs; specifically, whether this review identifies any neuroimaging studies that assist in elucidating this matter.

## Methods

### Search strategy

This review was conducted according to the Preferred Reporting Items for Systematic Reviews and Meta-Analyses (PRISMA) guidelines for systematic reviews [29]. The final database searches were conducted on 14 February 2017 and all relevant articles published up until this time were considered against the eligibility criteria outlined below, with no restriction on publication date to maximise results. Key search terms included: eating disorder, bulimi* (bulimia, bulimic), bing* (binge, bingeing), binge eating, MRI, fMRI, SPECT, PET, CT, neuro* (neurobiology, neuronal, neurotransmitters, neuroimaging, neuroscience). The precise search strategy developed for database searches is available on request from the authors.

The systematic review was conducted in four stages:

1. Developing inclusion and exclusion criteria for the database searches;
2. Searching selected electronic databases to identify papers meeting inclusion criteria for the review;
3. Study selection; and,
4. Appraisal and write-up of the studies that met inclusion criteria.

### Selection criteria

Potential peer-reviewed, published studies were identified using four electronic databases: PubMed, PsycInfo, Medline and Web of Science. Additional manual searches were conducted through reference lists. Studies were included if they (a) were an original research paper written in English; (b) studied humans only; (c) used samples of participants with clinician-diagnosed BN or BED; (d) recruited a HC sample with no eating disorder pathology and for studies including BED participants, a healthy, non-overweight control group was recruited; and (e) reported group comparisons between clinical and HC groups. Studies where participants were from adolescent or adult samples were included to broaden the number of possible studies in the review.

### *Inter-rater reliability*

Due to the high number of studies screened by abstract (n=186), a random subset was screened by two co-authors (ST, AB) to establish inter-rater reliability of the selection criteria. Excellent inter-rater reliability was established (ST: k = 0.769, *p* = 0.001), (AB: k = 0.880, *p* = .000).

### Data extraction and quality assessment

As there is no standardised criterion for the quality assessment of neurobiological studies, a modified version of the Downs and Black [30] Quality Index, adapted by Ferro and Speechley [31], was used. The Ferro and Speechley [31] index consists of 15 items of the original 27-item scale and is scored dichotomously as 0 (no/unable to answer) or 1 (yes). The index has four subscales: reporting, external validity, internal validity, and power. However within the Ferro and Speechley [31] index, some items were not applicable to the quality assessment of neurobiological studies. For this reason, four items were removed (response rate; estimates of the random variability; were staff / places / facilities where patients were studied representative of the treatment the majority of patients receive; were outcome measures valid and reliable) to prevent the neurobiological studies receiving an artificially low rating. Lastly, five additional items relevant to neurobiological studies of eating disorder patients (controlled for age; controlled for BMI; controlled for handedness; satiety pre-testing; and menstrual status) were added by the authors of this systematic review after consulting with experts in the field i.e. Psychiatrists specialising in the treatment of eating disorders; Staff Specialist Endocrinologists specialising in inpatient medical treatment for people with severe eating disorders. This created a new modified quality assessment scale for this systematic review consisting of 16 items. Quality ratings of the articles that were included in this systematic review ranged between seven and 11 out of a maximum score of 16. The majority (n=18) of the papers scored 9 or more out of 16.

The primary author conducted this modified quality assessment on all studies that met inclusion criteria for this review. To control for bias, a second author (AB) assessed 20% of the included studies using the same quality assessment. Agreement was reached over the methodology of the quality assessment and disparate ratings were discussed in two meetings. Excellent inter-rater reliability was established; Cohen’s kappa scores across the sub-set of studies ranging from (k = 0.871, *p* < 0.001) to perfect agreement (k = 1.0, *p* < .001).

Following the protocol of McClelland and colleagues [32] of a systematic review examining neuroimaging and behavioural studies, data were extracted from the included studies and summarised in a table that was then checked by two other reviewers. The data related to participants (age, female/male ratio); eating disorder diagnoses; sample size; procedure, including any task completed during neurobiological tests; exclusions; and brief findings. Due to the breadth of methodologies of the studies that met selection criteria, a narrative synthesis was then conducted, grouped according to the type of neuroimaging test and/or method used.

## Results

### Search results and selection of studies

**Fig. 1:**
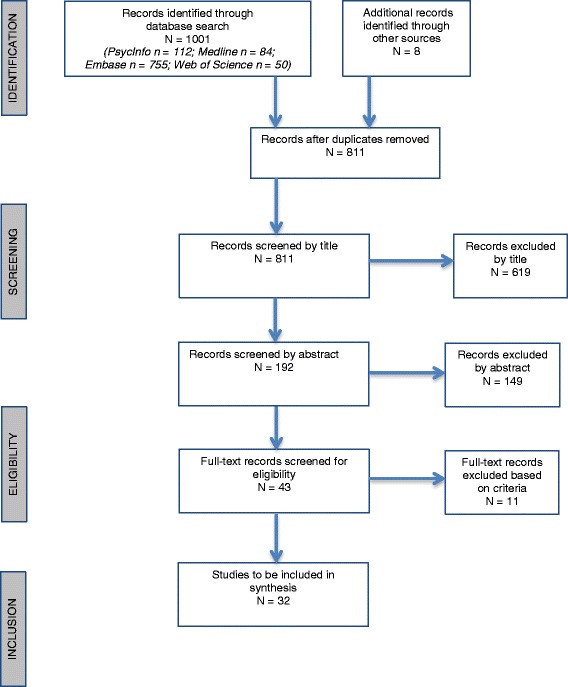
PRISMA flow chart of study identification and inclusion

An initial 1,003 studies were identified using the search strategy described above and 811 of these were screened by title after duplicates were removed. A total of 192 abstracts were screened and 149 studies were excluded at this point. This left 43 studies screened by full-text, with 11 studies excluded at this juncture. The final number of studies included in this review was 32. The main reasons for studies not meeting the criteria were: not including a BN or BED sample; not including a HC sample or only including an overweight control sample for BED studies; using a recovered clinical population where participants had been symptom-free for over 12 months; or, using neuropsychological measures (e.g. Stroop test) [33] but not neurobiological measures (e.g. fMRI). The diversity in the methodologies within the studies included in this systematic review precluded the completion of a meta-analysis. Figure 1 presents the PRISMA flowchart describing the inclusion and exclusion process.

### Study characteristics

A total of *n*= 32 studies met the inclusion criteria for this review. Sixteen studies on participants with BN, eleven studies on participants with AN and BN, three studies on participants with BN and BED and two studies on participants with BED were selected for review. The majority of included studies (n=21) used fMRI. Seven used MRI, with one of these studies using both MRI and Positron Emission Technology (PET). Of the remaining four studies, three utilized Single-Photon Emission Computed Tomography (SPECT) and one used PET. All but two of the included studies recruited female eating disorder patients only; one study recruited both men and women [23] and one study did not report this [34]. Sample sizes were relatively small; see below for details of sample sizes grouped according to the type of neurological test performed.

### Structural differences: Summary of MRI studies

MRI produces three-dimensional, anatomical images but cannot measure metabolic rates, unlike PET or SPECT. Seven studies using MRI to investigate structural differences between BN, BED and HCs met inclusion criteria. Sample sizes of MRI studies were consistently small; BN median and range: 10 (8-21); HC: 11 (7-21); for BED there was only one study, (*n*=17). See Table 1 for the extracted data from these studies. Earlier studies tended to examine structural differences or

**Table 1:** Characteristics and key findings of included studies using MRI as the primary method

Authors & Journal	Participants	Mean age (SD)	% Female	Procedure	Psychiatric / other exclusions	Findings
1. Coutinho et al. (2015) [22] *International Journal of Eating Disorders, 48*(2): 206-214.	BN (n=21) HCs (*n*=20)	BN: 31.57 (8.27) HCs: 30.9 (8.79)	100%	One resting-state MRI	Substance abuse disorder; suicidal ideation; Axis I disorder other than eating disorder; psychotropic medication with the exception of anxiolytics and antidepressants	Volume reduction in the CN within the frontostriatal circuit in BN compared to HCs
2. Doraiswamy et al. (1990) [37] *Biological Psychiatry, 28:* 110-116.	BN (*n* = 10) AN (*n* = 8) HCs (*n* = 13)	BN: 24 (2.5) AN: 22.8 (4.4) HCs: 27.5 (5.1)	100%	One resting-state MRI	Major affective disorder	AN & BN vs HCs: smaller pituitary gland area and heights A trend approaching statistical significance was found: the area of the pituitary was negatively correlated with duration of illness
3. Galusca et al. (2014) [39] *The World Journal of Biological Psychiatry, 15*: 599-608.	BN-P* (n=9) HCs (n=11) *only *severe* BN-P participants selected; criterion being at least one binge-purge episode/day for at least six months	BN-P: Only the age range (18-30y) was reported. No mean or SD. HCs: No data, however reported to be age-matched.	100%	MRI and PET completed 2h following lunch + PET to specifically examine serotonergic activity / binding potential of [^18^F]MPPF (a serotonin specific radiogland used in PET capable of assessing change in brain serotonin)	Chronic or congenital disease; alcohol, tobacco or drug consumption; previous or current diagnosis of AN-R; medication In the BN group: oral contraceptive pill	BN vs HCs: increased binding potential in four clusters in the brain: Insula and transverse temporal cortex, operculum, temporo-parietal cortex Abnormalities in impaired activation, glucose metabolism or ligand binding in areas including insula and temporal parietal cortex, hippocampal region, inter-hemispheric cortex, PFC and dorsal raphe nucleus
4. Hoffman et al. (1989) [35] *Biological Psychiatry, 25:* 894-902.	BN (*n*=8) HCs (*n*=8)	BN:24.1 HCs: 26.8 No SD reported	100%	One resting-state MRI	Past diagnosis of AN; current diagnosis of major affective disorder; alcohol abuse	BN vs HCs: cortical atrophy found in the sagittal cerebral / cranio ratio (SCCR) but not in the ventricle:brain ratio (VBR) Significant positive correlation between binge frequency and VBR
5. Hoffman et al. (1990) [38] *Biological Psychiatry, 27*: 116-119.	BN (*n*=8) HC (*n*=7)	BN 24.3 (3.2) HC 24.3 (3.4)	100%	One resting-state MRI	Current diagnosis of major affective disorder; alcohol abuse In the BN & HC group: lifetime diagnosis of AN; medication	BN vs HC: Significant decrease in inferior frontal grey matter
6. Husain et al. (1992) [36] *Biological Psychiatry, 31*: 735-738.	BN (*n*=12) AN (*n*=12) HCs (*n*=11)	BN 24.5 (4) AN: 25.3 (7) 27.8 (6)	100%	One resting-state MRI	In the BN group: past diagnosis of AN	AN vs. BN & HCs: Significantly smaller thalamus and midbrain (mesencephalon) area The ratio of thalamus to cerebral hemisphere and midbrain to cerebral hemisphere was significantly smaller in BN & AN vs. HCs however post-hoc tests showed this result was only related to AN participants
7. Schäfer et al. (2010) [24] *NeuroImage, 50*: 639-643.	BN-P (*n*=14) BED (*n*=17) HCs (n=19)	BN-P: 23.1 (3.8) BED: 26.4 (6.4) HCs: 22.3 (2.6)	100%	One resting-state MRI to examine structural brain abnormalities. Grey matter volumes (GMV) for specific regions involved in food / reinforcement processing were analysed via voxel-based morphometry: medial / lateral OFC, insula, ACC, ventral / dorsal striatum	Depression; left-handedness; medication	BN vs. BED: greater GMV of medial and lateral orbitofrontal cortex as well as ventral & dorsal striatum BN vs HCs: increased GMV of medial OFC & ventral striatum BED vs HCs: greater GMV of ACC & medial OFC BN & BED vs. HCs: greater volumes of the medial OFC BN vs BED & HCs: increased ventral striatum volumes; BMI was negatively correlated with striatal grey matter volume while purging was positively correlated with ventral striatum volume

volumetric differences between certain cerebral structures compared to the overall brain using a ratio calculation. Hoffman et al. [35] published one of the first neurobiological studies of BN patients; a resting-state MRI with BN and HC participants to assess cerebral atrophy. It was found that the BN group had a significantly lower sagittal cerebral:cranial ratio however there was no difference between the two groups on the ventricle:brain ratio [35]. In a similar study, Husain et al. [36] reported the ratio of thalamus:cerebral hemisphere and midbrain:cerebral hemisphere was significantly smaller in AN participants but not in BN or HC participants. Doraiswamy et al. [37] used MRI to investigate pituitary differences in AN and BN participants compared with HC subjects; results demonstrated the overall area and height of the pituitary was significantly smaller in eating disorder participants compared to the control group. Several groups reported grey matter reduction. Hoffman et al. [38] reported a significant decrease in the inferior frontal grey matter of BN participants compared to HCs. Coutinho and colleagues [22] reported a similar finding of reduced CN volume in BN participants, after conducting a resting-state MRI with BN and HC participants.

In the only study to use MRI and PET together, Galusca and colleagues [39] investigated cerebral serotonergic activity in severe BN-Purging (BN-P) (where severe BN-P was defined as at least one binge-purge episode every day for at least six months) and HC subjects two hours after lunch. Widespread abnormal cerebral serotonergic activity characterized the BN group, however inter-individual heterogeneity was found when individual comparisons were conducted between each BN patient and the HC group. This ranged from isolated to widespread increases in the binding potential of [^18^F]MPPF (a serotonin specific radiogland used in PET). Binding potential is the central measure of PET [39]. Finally, Schafer and colleagues [24] conducted MRI with BN, BED and HC participants to investigate grey matter volume (GMV) abnormalities. The authors used voxel-based morphometry to analyse specific brain regions known to be involved in food and reinforcement processing (medial and lateral orbito-frontal cortex [OFC], insula, anterior cingulate cortex and ventral / dorsal striatum). The BN and BED participants had greater GMV in the medial OFC compared to HC participants. The BN group also had significantly increased ventral striatal volumes, part of the neural reward system, when compared to the BED and HC groups [24].

### Functional differences: Summary of fMRI studies

fMRI provides good coverage and excellent spatial resolution, relying on the interrelationship between cerebral blood flow, energy demand and neural activity [40]. The primary fMRI neuroimaging signal most researchers examine is the blood oxygen level-dependent (BOLD) contrast signal, which provides a reliable measure of a local increase in neural activity [40]. Twenty-one studies using fMRI as the primary neurological test met inclusion criteria for this review [18, 23, 25, 26, 41–57]. Eleven studied participants with BN [18, 25, 26, 43, 45–47, 50, 51, 53, 58], seven examined participants with BN and AN [42, 48, 49, 54–57, 59], two examined participants with BN and BED [44, 52] and one study examined participants with BED [23].

Overall, fMRI studies had marginally higher sample sizes than for the MRI studies reported above; BN median and range: 13 (8-32); HC: 19 (12-34); BED: only three studies included BED participants hence the median could not be calculated, range (12-19). See Table 2 for data extracted from these studies. As can be seen in Table 2, there is obvious heterogeneity across the design of the included fMRI studies. For this reason, fMRI results have been grouped according to the type of stimuli used during testing (decision making and learning paradigms; food-related stimuli; body image-related stimuli).

**Table 2:** Characteristics and key findings of included studies using fMRI as the primary method.

Authors & Journal	Participants	Mean age (SD)	% Female	Method	Psychiatric / other exclusions	Findings
1. Amianto et al. (2013) [57] *Cerebellum, 12:* 623-631.	AN (*n*=12) BN (*n*=12) HC (*n*=10)	AN: 20(4) BN: 23(5) HC: 24(3)	100%	One resting-state fMRI.	Lifetime history of psychosis, schizophrenia, schizoaffective disorder, delusional disorder, bipolar I/II disorder, psychotic depression, organic mood disorder; severe medical illness; severe underweight that could not be managed as an outpatient; use of psychotropic medication; neurological disease	AN vs BN & HC: grey matter reduction AN & BN vs HC: hyperconnectivity of the cerebellar network to the parietal cortex; increased bilateral connectivity of cerebellar ICN with temporal poles BN vs AN & HCs: GMV reduction in the CN
2. Balodis et al. (2013) [23] *Biological Psychiatry, 73*:877-886.	Obese BED (*n*=19) Obese non-BED (*n*=19) HCs (*n*=19)	BED 43.7 (12.7) OB 38.3 (7.5) HC 34.8 (10.7)	BED 73.7% OB 52.6% HC 52.6%	fMRI completed while completing MIDT (monetary incentive delay task)	In the obese non-BED or HC group: past history of, or current binge eating or other eating disorder diagnosis	Anticipation processing: BED vs OB: decreased ventrostriatal (VS) and striatal activity; OB vs HCs: increased VS activity Outcome processing: BED vs OB & HCs: diminished activity in PFC and Insular
3. Bohon & Stice (2011) [58] *International Journal of Eating Disorders, 44*(7): 585-595.	BN sub-threshold* (1xBE & comp/wk) (*n*=11) BN (*n*=2) HCs (*n*=13) * (Sub-BN = 1 x binge episode/week – sub-threshold for DSM-IV criteria, however this frequency meets the new DSM-V criteria for BN)	Not reported per group. For all participants: 20.3 (1.87)	100%	fMRI examining reward circuitry during actual (choc milkshake) and anticipated (tasteless solution) food intake	Any Axis I disorder; food allergy to milkshake / taste aversion to chocolate milkshake In the BN group: psychoactive medications other than SSRIs (sertraline & fluoxetine)	BN vs HCs: less activation in right precentral gyrus in both anticipatory and consumatory conditions; less activation in right anterior insula while anticipating the milkshake; and less activation in the left middle frontal gyrus, right posterior insula, left thalamus in response to milkshake
4. Brooks et al. (2011) [42] *PLoS One, 6*(7):e22259.	BN (*n*=8) AN-R (*n*=11) AN-BP (*n*=7) HCs (*n*=24)	BN: 25 (7.1) AN: 26 (6.8) HC: 26 (9.5)	100%	fMRI while asking participants to imagine eating the foods shown in photographs (72 colour photos of high and low energy, sweet & savoury foods; 72 photos of non-food items	Left handedness; caffeine / alcohol within specified times preceding the fMRI; history of head trauma, hearing or visual impairment, neurological disease In the ED groups: psychotropic medications other than SSRIs	In response to food vs. non-food images: BN vs HCs and AN: greater activation in visual cortex, right dorsolateral prefrontal cortex, right insular cortex and precentral gyrus BN vs. HC: deactivation in bilateral superior temporal gyrus, insular cortex, visual cortex BN vs AN: reduced activation in parietal lobe, dorsal posterior cingulate cortex BN vs AN-R: increased activation in bilateral inferior temporal lobe, left visual cortex, posterior cingulate & left inferior parietal lobe and deactivation in right precentral gyrus BN vs AN-BP: greater activation in the left cerebellum, left parahippocampal gyrus, left posterior cingulate cortex, right supplementary motor area, and deactivation in left inferior temporal gyrus
5. Celone et al. (2011) [25] *NeuroImage, 56:* 1749-1757.	‘Sub-threshold’* BN (*n*=18) HCs (n=19) * (Sub-BN = 1 x binge episode/week – sub-threshold for DSM-IV criteria, however this frequency meets the new DSM-V criteria for BN)	Sub-BN: 20.67 (2.10) HC: 20.42 (1.95)	100%	fMRI during Weather Prediction Task (WPT), a probabilistic learning paradigm.	Previous or current neurological or medical disease; learning disability; substance abuse; history of significantly low body weight (<85% of ideal body weight); past or current AN	No behavioural differences in performance Results demonstrate processing inefficiencies in the fronto-striatal system in BN. BN women demonstrated increased overall category learning-related activity in the right caudate nucleus and bilateral dorsolateral PFC and decreased suppression of the category learning related BOLD signal in the anterior cingulate cortex. The direction of the BOLD signal changes within the fronto-striatal system differs from the initial hypothesis
6. Cyr et al. (2016) [43] *Journal of the American Academy of Child and Adolescent Psychiatry, 55*(11): 963-972.	BN (*n*=27) HCs (*n*=27)	BN: 16.6 (1.5) HC:16.3 (2.1)	100%	fMRI BOLD response during reward based spatial learning task (virtual learning)	History of neurological illness; past seizures; head trauma with loss of consciousness (LOC); mental retardation; pervasive developmental disorder; current Axis I disorder (other than depressive / anxiety disorder for clinical group)	BN vs HCs: engaged the right anterior hippocampus when receiving *unexpected* rewards HCs vs BN: engaged the right IFC when searching spatially and the right anterior hippocampus when receiving *expected* rewards Overall the data suggest abnormal functioning of the anterior hippocampus and fronto-striatal circuits during reward-based spatial learning Clinical correlates: Severity of BN was significantly associated with activation of the right anterior hippocampus during reward processing
7. Lee et al. (2017) [44] Neuroscience Letters, *accepted manuscript 26/04/17*	BN (*n*= 13) BED (*n*=12) HC (*n*=14)	BN: 23.7 (2.2) BED: 23.6 (2.6) HC: 23.3 (2.2)	100%	fMRI performed while participants completed the Stroop match-to-sample task, in which participant attention is controlled by an interaction between bottom-up sensory processing and top-down cognitive processing driven mainly by the prefrontal cortex. The task was modified to include food and non-food conditions.	BMI < 17.5; current or past psychiatric disorder; traumatic brain injury; neurological illness; current or past use of psychiatric medications	BN vs HC: lower accuracy indicating impaired cognitive control over interference. Higher activation in the premotor cortex and dorsal striatum in response to food images BED vs HC: higher activation in the ventral striatum in response to food images
8. Marsh et al. (2009) [45] *Archives of General Psychiatry, 66*(1): 51-63.	BN (*n*=20) HCs (*n*=20)	BN: 25.7(7.0) HC: 26.35(5.7)	100%	fMRI used to examine BOLD during performance on a Simon spatial incompatibility task (SSIT). Two groups compared on patterns of brain activation.	History of neurological illness; past seizures; head trauma with LOC; mental retardation, pervasive developmental delay In the BN group: current Axis I disorder excluding major depression	BN vs HC: responded significantly more impulsively and made a greater number of errors on the SSIT BN group: The number of objective binge episodes correlated inversely with the significantly increased activation of the right medial prefrontal, temporal and inferior parietal cortices HC vs BN: greater activation in the anterior cingulate cortex during incorrect responses and activated the striatum more when responding incorrectly
9. Marsh et al. (2011) [46] *American Journal of Psychiatry, 168*(11): 1210-1220.	BN (*n*=18) HCs (*n*=18)	BN: 18.4 (2.1) HC: 17.3 (2.4)	100%	fMRI used to examine BOLD during performance on a Simon spatial incompatibility task. Two groups compared on patterns of brain activation.	History of neurological illness; past seizures; head trauma with LOC; mental retardation, pervasive developmental delay In the BN group: current Axis I disorder excluding major depression	BN and HCs performed comparably however during correct responses in conflict trials the frontostriatal circuits failed to activate to the same degree in the BN group BN vs HC: demonstrated abnormal patterns of activation in the frontostriatal and ‘default mode’ systems; specifically they did not have the same magnitude of activity in the frontostriatal circuits known to underlie self-regulatory control, including the right inferior frontal gyrus, dorsolateral PFC, and putamen
10. Marsh et al. (2015) [47] *Biological Psychiatry, 77:* 616-623.	BN adolescent <19yo (n=16) BN adult (n=16) HCs (*n*=34)	Not reported for either group; only that there were adolescents and adults in both BN & HCs.	100%	fMRI completed to compare morphological characteristics of their cerebral surface	History of neurological illness; past seizures; head trauma with LOC; mental retardation, pervasive developmental delay In the BN group: current Axis I disorder excluding major depression	BN vs HCs: Significant reduction of local volumes on brain surface found in the frontal and temperoparietal areas (bilateral middle frontal and precentral gyri; right postcentral gyrus and lateral superior, and lateral superior and inferior frontal gyri of the left hemisphere). Reductions were also found in temperoparietal regions including bilateral inferior temporal gyri, right superior parietal gyrus and cuneus, bilateral posterior cingulate cortices, left precuneus and fusiform gyrus. Enlargements detected in the bilateral middle/inferior occipital and lingual gyri and right inferior parietal lobule in the BN group. Significant inverse associations reported between cerebral surface morphology and objective binge and vomiting episodes in the bilateral IFG, PreCG and PoCG.
11. Miyake, Okamoto, Onada, Kurosaki et al., (2010) [59] *Neuroimaging, 181*: 183-192.	BN (*n*=11) AN-R (*n*=11) AN-BP (*n*=11) HCs (*n*=11)	BN: 24.5 (5.8) AN-R: 22.2 (4.1) AN-BP: 28.3 (4.5) HC: 26.5 (5.5)	100%	fMRI with emotional decision task with distorted body images (varying degrees of ‘thinness and fatness’ of own and healthy female body photo)	Presence of Axis I or II disorder other than ED; left handedness In the BN and HC groups: history of AN	In AN-R, AN-BP and HCs, but not BN, the amygdala was significantly activated in response to own ‘fat-image’ AN-BP and HCs vs BN and AN-R: medial PFC significantly activated AN-R vs other ED groups: amygdala significantly more activated in response to ‘fat’ image of another woman
12. Miyake, Okamoto, Onada, Shirao et al. (2010) [49] *Neuroimage, 50:* 1333-1339.	BN (*n*=12) AN-R (*n*=12) AN-BP (*n*=12) HCs (*n*=12)	BN: 25.0 (6.9) AN-R: 27.0 (9.0) AN-BP: 27.2 (4.8) HC: 25.4 (5.8)	100%	fMRI while completing emotional word decision making task, examining processing of words (negative body image words e.g.obesity; and neutral words).	Presence of Axis I or II disorder other than ED; left handedness *Note - one BN participant had a history of AN*	Negative body image words condition:AN-R & AN-BP vs BN & HC: right amygdala significantly more activated BN & AN-BP vs HC: left medial PFC significantly more activated AN-R & AN-BP vs HCs: left inferior parietal lobule significantly more activated Overall: results indicated that distorted cognition of negative body image words in eating disorder patients were related to enhanced activation in amygdala and mPFC. However there were no group differences in nonspecific negative emotion words
13. Mohr et al. (2011) [50] *NeuroImage, 56:* 1822-1831.	BN (*n*=15) HCs (*n*=15)	BN: 24.8 (3.2) HC: 25.5 (4.5)	100%	fMRI while rating satisfaction and size estimation of distorted own body photographs	History of substance abuse; schizophrenia and psychotic symptoms; bipolar disorder; neurological illness; closed head injury; left handedness	The activation pattern in the insula reflected satisfaction ratings of BN and HCs HCs vs BN: in terms of general differences in body image processing (not specifically during satisfaction / perception conditions), the MFG & right posterior parietal cortex demonstrated significantly greater activation (potentially reflecting a reduced spatial manipulation capacity) HC vs BN: during body size estimation/perception task, the MFG was significantly more activated in HCs than BN, and the MFG was recruited significantly more in the perception vs satisfaction task HCs vs BN: posterior temporal-occipital cortex was sensitive for body image distortion (this ‘type of modulation’ was not observed in BN) HC & BN: The amount of bilateral insula activity reflected the pattern of satisfaction rating task. In BN a linear trend occurred with a decline in insula and MFG activity from thinner to fatter images, although results weren’t as clear in HCs
14. Pringle et al. (2011) [51] *Neuropsychologia* *49*:3272-3278.	BN (*n*=11) HCs (*n*=16)	BN: 24.55 (4.97) HC: 27.38 (5.44)	100%	fMRI to examine self-referent emotional processing, where patients had to endorse 60 personality characteristic words as ‘me’ or ‘not me’ in rapid event related design.	Left handedness; medication	BN vs HCs: rating of negative personality descriptors was associated with reduced activity in the parietal, occipital and limbic areas, including the amygdala
15. Schienle et al. (2009) [52] *Biological Psychiatry, 65:* 654-661.	BED (*n*=17) BN-P (*n*=14) HCs normal weight (n=19) Controls - overweight (C-OW) (*n*=17)	BED: 26.4 (6.4) BN-P: 23.1 (3.8) HC-N: 22.3 (2.6) HC-O: 25.0 (4.7)	100%	fMRI completed after 12-hr overnight fast, while participants viewed three categories of images: high calorie (e.g. ice cream, french fries), disgust- inducing (e.g. dirty toilets, maggots) and affectively neutral (e.g. household items).	Medication; clinically relevant depression; left handedness	All participants demonstrated increased activation in the OFC, ACC and insula highlighting a basic appetitive response pattern. No group differences in disgust-inducing images BED vs. all other groups: enhanced reward sensitivity, stronger medial OFC activity while viewing food images BN vs. all other groups: greater ACC activation and insula activation while viewing food images
16. Seitz et al. (2016) [18] *European Child and Adolescent Psychiatry, 1*: S185-203.	BN (*n*=20) HCs (*n*=20)	BN: 18.71 (2.53) HC: 17.90 (1.35)	100%	fMRI while participants completed a modified version of the Attention Network Task (ANT), investigating neural networks associated with alerting, reorienting and executive attention.	History of psychosis; substance abuse; IQ <80	BN vs. HCs: • Higher ADHD scores, especially inattention. • Hyperactivation in the parieto-occipital regions and reduced deactivation of the precuneus, part of the default-mode-network areas during ‘alerting’ • Posterior cingulate activation during alerting correlated with severity of BN symptoms • Exploratory correlation analyses found significant associations between neural activity in the ‘alerting’ condition in the bilateral middle cingulate and global eating disorder symptoms; significant inverse correlation between activity in the temperoparietal junction and ADHD symptoms; and activity in the right parahippocampus was inversely correlated with impulsivity scores
17. Skunde et al. (2016) [26] *Journal of Psychiatry and Neuroscience, 41*(5): E69-E78.	BN (*n*=28) HCs (*n*=29)	BN: 27.54 (10.52) HC: 27.25 (6.68)	100%	fMRI while completing a general and food-specific (participants selected 8 of their favourite food images from a set of 85 high-calorie foods prior to completing the task) no-go task (the no-go task is a sub-task of the go-no-go task and measures behavioural inhibition).	Biploar disorder; psychosis; history of head injury; neurologic disorder; diabetes mellitus; nicotine / drug / alcohol abuse; lifetime diagnosis of BPD In HCs: current psychotropic meditation In BN: medication other than antidepresants	BN vs. HCs: reduced activation in the right sensorimotor area (postcentral gyrus, precentral gyrus) and right dorsal striatum (caudate nucleus, putamen) HC vs BN (high frequency BEs only): stronger activation in the right postcentral gyrus, right caudate nucleus and right putamen
18. Spangler et al. (2012) [53] *International Journal of Eating Disorders, 45*(1): 17-25	BN (*n*=12) HCs (*n*=12)	BN: Age range reported only (18-38) HC: (18-30)	100%	fMRI while looking at computer- generated images of ‘thin’ (BMI= 18) or ‘fat’ (BMI=31) bodies (and control condition: scrambled image). Participants instructed *to imagine someone is comparing your body to the body of the woman in the picture.*	In BN: medication other than antidepressants	BN: no significant difference found in brain activation while looking at thin vs fat images BN vs. HC: mPFC activation was significantly greater while viewing ‘fat’ images, with increased activity in the regions associated with emotional processing. No differences between groups in the thin condition In the mPFC, the peak location of activation for BN patients was in the pgACC rather than dorsal mPFC, as it was for HCs
19. Uher et al. 2004 [54] *American Journal of Psychiatry, 161*(7): 1238-1246	BN (*n*=10) AN (*n*=16) HCs (*n*=19)	BN: 29.80 (8.80) AN: 26.93 (12.14) HC: 26.68 (8.34)	100%	fMRI completed while being presented with photographs of savoury and sweet foods; non-food items; emotionally aversive photographs and neutral stimuli.	Axis I disorders other than ED; neurological or psychiatric illness aside from ED; psychotropic medication other than antidepressants	BN vs HCs: greater occipital and cerebellar activity BN vs AN & HCs: decreased activation in the anterior and lateral PFC in response to food images (associated with suppressing unwanted behaviours) AN & BN vs HC: significantly increased medial PFC reaction to food images
20. Uher et al. 2005 [55] *Biological Psychiatry, 58*(12):990-997	BN (*n*=9) AN (*n*=13) HCs (*n*=19)	BN: 29.6 (9.3) AN: 25.4 (10.2) HC: 26.6 (8.6)	100%	fMRI to examine cerebral correlates of body image activity when participants looking at line drawings of underweight (BMI = <17.5), normal weight (20<BMI<25), and overweight (BMI 27.5) female bodies vs. control images (line drawings of houses)	Psychosis; alcohol or drug dependence; neurological or psychiatric illness aside from ED; psychotropic medication other than antidepressants	No regions of significantly increased activations in either eating disorder group, compared to the control subjects Across AN, BN & HCs, the lateral fusiform gyrus, inferior parietal cortex and lateral PFC were activated in response to body shapes vs. control condition
21. Vocks et al. 2010 [56] *Journal of Psychiatry and Neuroscience, 35*(3): 163-176.	AN (*n*=13: 8 AN-R and 6 AN-BP) BN (*n*=15) HC (*n*=27)	AN: 29.08 (9.79) BN: 28.4 (7.07) HCs: 26.74 (7.6)	100%	fMRI while participants looked at 16 standardised photographs of their own body and another woman’s body (BMI 19), taken while wearing a bikini.	Left handedness; personality disorder	AN & BN vs. HCs: while viewing photographs of their own body, eating disorder patients showed weakened activity in the left inferior parietal lobule AN vs. BN & HCs: higher amygdala activity while looking at photographs of another woman’s body AN vs. BN & HCs: significantly greater activation in the bilateral superior temporal gyrus

#### Non-food decision making and learning paradigms

Six papers in this review utilized decision making or learning paradigms during fMRI. Balodis et al. [23] found obese BED patients had decreased VS activity during *anticipation* processing while completing a monetary incentive delay task, however during *outcome* processing the group demonstrated diminished prefrontal cortex (PFC) and insula activity. Using a probabilistic learning paradigm (Weather Prediction Task), sub-threshold BN patients demonstrated hyperactivity and overall processing inefficiencies in the frontostriatal system [25]. A similar finding was reported by Cyr et al [43], who used a reward-based virtual learning task, with abnormal functioning of the anterior hippocampus and frontostriatal circuits reported in BN compared to HC participants. In the same study, BN symptom severity was significantly associated with the activation of the right anterior hippocampus during reward processing. Two studies [45, 46] used the Simon Spatial Incompatibility Task to examine BOLD during fMRI. In contrast to Celone et al. [25], Marsh et al. [46] found hypoactivity in the frontostriatal circuits known to contribute to self-regulatory control, including the right inferior frontal gyrus, dorsolateral PFC and putamen, in BN compared to controls. Finally, Seitz et al. [18] reported a range of differences in the neural networks associated with alerting, reorienting and executive attention between BN and HC participants, who underwent fMRI while completing a modified Attention Network Task (ANT), as well as a range of correlations between neural activity, BN symptoms and scores on a measure of impulsivity associated with attention deficit hyperactivity disorder (ADHD).

#### Food-related stimuli

Six of the fMRI studies in this review used food-specific stimuli; most of these used photographs of food items [26, 42, 52, 54, 58], however one study used actual food stimulus of 0.5mL portions of chocolate milkshake [58], and one study asked participants to think about eating the food shown to them in photographs [42]. In the first study to examine neural reward circuitry in BN in response to actual and anticipated food intake [58], decreased but not significantly different activation was found in the right precentral gyrus during both anticipating and consuming chocolate milkshake, and decreased activation in the left middle frontal gyrus, right posterior insula and left thalamus, compared to the HC group. The precentral gyrus is the site of the primary motor cortex while the insula has a central role in gustatory and hedonic taste processing and alongside the middle frontal gyrus, it is activated in response to pleasurable taste [58, 60, 61]. Brooks et al [42] compared BN, AN and HC participants’ neural responses to images of high- and low-energy foods versus non-food items. In response to food images, the BN group demonstrated increased activation in the visual cortex, right dorsolateral prefrontal cortex, right insular cortex and precentral gyrus. Relative to the HC group, the BN group also showed deactivation in the bilateral superior temporal gyrus/insula and visual cortex, and compared to the AN group, had deactivation in the parietal lobe and dorsal posterior cingulate cortex and increased activation in the caudate, superior temporal gyrus, right insula and motor area [42].

The remaining studies utilizing food images reported a range of both decreased and increased neural activation in the binge eating groups compared to AN and HC participants. Schienle and colleagues [52] reported increased insula activation in women with BN in their study using images of high-calorie foods, disgust-inducing and neutral images during fMRI following an overnight fast, compared to BED and HC groups. Food images were experienced positively across all groups with increased activation of the orbitofrontal cortex, anterior cingulate cortex and insula and there were no group differences in neural response to viewing disgust-inducing images. Women with BED demonstrated greater medial orbitofrontal response while viewing food images compared to BN and HC groups, while the BN group demonstrated greater anterior cingulate cortex and insula activation [52]. In a study examining neural response to food images used as interference during the Stroop task [33] (a neuropsychological test of self-regulation utilising the frontostriatal region), women with BED had stronger activation in the VS, while women with BN had greater activation in the premotor cortex and dorsal striatum [44].

Skunde et al. [26] personalized visual food stimuli for BN participants, by asking them to select eight favourite food images to use during a measure of behavioural inhibition, finding the BN group had reduced motor cortex activation including the primary motor, premotor and primary somatosensory cortices; and reduced activation in the right sensorimotor area (postcentral and precentral gyrus) and right dorsal striatum (caudate nucleus, putamen), relative to HCs. Importantly, these results suggest diminished frontostriatal and sensorimotor control contribute to diminished inhibitory control in BN. Finally, Uher et al. [54] found women with BN had decreased activation in the anterior and lateral prefrontal cortex in response to viewing food images compared to AN and HC groups, and greater occipital and cerebellar activity compared to the HC group.

#### Body image-related stimuli

Body image distortion is a key symptom of eating disorders. Six studies included in the review used body image-related photographs, images or tasks during fMRI [48–50, 53, 55, 56]. As in the other categories of fMRI studies, there was heterogeneity in the stimuli used. One study used negative body-related words e.g. obesity [49], reporting distorted cognition of negative body image words in women with BN and AN may be linked to increased activation in the amygdala and medial prefrontal cortex (mPFC). Two of the studies used real and distorted photographs of participants’ own bodies and control bodies. Miyake, Okamoto, Onada, Kurosaki et al. [48] reported the amygdala and mPFC were significantly activated in AN and HC women in response to their own ‘fat’ distorted image however this wasn’t found in BN women. Mohr and colleagues [50] used similar stimuli and reported that BN participants did not recruit the middle frontal gyrus (MFG) compared to HCs when estimating the size of their bodies, possibly reflecting reduced spatial manipulation. Increased activity in the lateral occipital cortex was sensitive for body size distortions in the control group but not in the BN group and the authors proposed the pattern of results may underlie body size overestimation in BN [50]. Interestingly, in the BN group a linear trend was observed, wherein insula and MFG activity declined as body photographs moved from thinner to actual and fatter images [50]. In a related study, Vocks et al. [56] used standardized photos of women’s own bodies and another woman’s body wearing a bikini, finding increased activation in the left middle temporal gyrus and middle frontal gyrus in BN and AN groups. Two studies [53, 55] used non-naturalistic stimuli. Spangler et al. [53] examined the activity of the mPFC, associated with self-referencing, during fMRI while participants imagined their bodies being compared to computer-generated ‘thin’ (Body Mass Index [BMI] =18) and ‘fat’ (BMI = 31) images. Women with BN experienced significantly greater mPFC activation when viewing the overweight female image compared to HCs, while no differences were found between groups when viewing the thin female image. Lastly, Uher et al. [55] used simple line drawings of underweight (BMI = <17.5), normal weight (20 < BMI < 25) and overweight (BMI = >27.5) bodies and found no regions of significantly increased activation in women with BN or AN compared to the HCs, however reported the patient group as a whole demonstrated weaker occipitotemporal cortex and parietal cortex activation to body shapes compared to HCs.

#### fMRI studies – other

Three fMRI papers that did not fit into the previous categories are reviewed here. Amianto and colleagues [57] conducted one resting-state fMRI in AN and BN groups, finding grey matter reduction and decreased connectivity of the cerebellar network to the parietal cortex and increased bilateral connectivity of intrinsic connectivity networks (ICNs). The BN group, when compared to the AN and HC groups, demonstrated grey matter reduction in the caudate nucleus (CN), part of the dorsal striatum and frontostriatal circuit. Marsh and colleagues [47] assessed morphological measures of the cerebral surface and compared to HCs, the BN group had significant reductions of local volume on the brain surface in the frontal and temperoparietal areas [47]. The local volume reduction in the inferior frontal regions was inversely correlated with age, symptom severity and pre-fMRI performance on the Stroop test. Pringle et al. [51] used a self-referent emotional processing task in BN, wherein participants had to rapidly endorse 60 personality characteristic words as either ‘me’ or ‘not me’. The BN group experienced decreased activity in the parietal, occipital and limbic areas when processing negative personality descriptors compared to HCs [51].

#### Functional differences: Summary of SPECT and PET studies.

Three studies met inclusion criteria for this systematic review using SPECT, which provides a functional, quantitative measure of regional cerebral blood flow (rCBF), a metric of brain function [62]. Two studies in this review used PET; one was described earlier that used combined MRI and PET [39], and the other described here using only PET. PET provides a measure of glucose metabolism rates in the brain, also a measure of brain function. Sample sizes were extremely small in this category of papers, relative to MRI and fMRI studies; BN median and range: 8 (5-21); HC:11.5 (9-12); BED: only one study included participants with BED (n=8). See Table 3 for the data extracted from these studies.

**Table 3:** Characteristics and key findings of included studies using SPECT and PET as the primary method.

Authors & Journal	Participants	Mean Age (SD)	% Female	Procedure	Psychiatric / other exclusions	Findings
1. Beato-Fernandez et al. (2011) [34] *Actas Espanolas de Psiqiuiatria, 39*(4): 203-10.	AN-R (*n*=11) AN-P (*n*=10) BN-NP (n=7) BN-P (*n*=14) HCs (*n*=12)	AN-R: 27.1 AN-P: 28.4 BN-P: 30.7 BN-NP: 34.7 HC: 20.6 *No SD of mean age reported*	Not reported	3x SPECT scans to measure rCBF during rest condition; calm visual stimulus condition and another after seeing their own body (filmed).	Left handedness; psychiatric illness aside from ED; neurological disorders	AN-R, AN-P, BN-P, BN-NP vs HCs: decreased right temporal rCBF when moving from rest condition to neutral visual image AN-R & BN-P vs other groups: increased right temporal rCBF going from neutral visual image to own body visual image
2. Karhunen et al. (2000) [63] *Psychiatry Research: Neuroimaging Section, 99*: 29-42.	OB BED (*n*=8) OB non-BED (n=11) HCs (*n*=12)	OB BED: 36.1(9.3) OB non-BED: 45.0 (10.0) HCs: 39.8(9.7)	100%	1 x SPECT scan to measure rCBF while participants were looking at a control image (landscape) and 1 x SPECT scan while participants were looking at a portion of real food after an overnight fast.	Left handedness; ‘No other disorders or medication known to affect the variables examined’ (pp31)	OB BED vs OB non-BED & HCs: significantly greater increase in rCBF in the left hemisphere compared to the right hemisphere, particularly in the frontal and prefrontal cortices, in the food exposure condition All groups experienced a significant increase in hunger in the food exposure condition. In the OB-BED group only, this was associated with significantly higher rCBF in the left frontal and pre-frontal cortices
3. Delvenne et al. (1997) [65] *International Journal of Eating Disorders, 21*(4):313-320.	BN (*n*=11) HCs (*n*=11)	BN: 26.2 (10.9) HCs: 25.7 (2.1)	100%	Resting state PET with (18-F) fluorodeoxyglucose used to evaluate cerebral glucose metabolism.	History of electroconvulsive therapy (ECT); significant abnormalities on physical and neurological examination; left handedness; no psychoactive medication for a minimum of 10 days; history of neuroleptic medication	BN vs HCs: absolute hypometabolism of glucose both globally and regionally, notably in the parietal and superior frontal cortices. The BN group also showed a lower relative regional cerebral glucose metabolism in the parietal cortex
4. Nozoe et al. (1995) [64] *Brain Research Bulletin, 36*(3): 251-255.	BN (*n*=5) AN (*n*=8) HCs (*n*=9)	BN: 21.0 (2.9) AN: 24.1 (7.8) HCs: 20.3 (1.0)	100%	Examined rCBF using SPECT before and after food intake (slice of cake)	Left handedness; abnormal neurological findings	BN vs AN & HCs: highest rCBF before eating in the left temporal and bilateral inferior frontal regions. Also, BN showed less increase in cortical activity post-eating BN and AN showed opposite patterns of frontal reaction to food stimuli AN: showed no marked cortical laterality or activation in any cortical area pre-eating but showed greater increased cortical activity post-eating

Two of the SPECT studies [34, 63] measured participants’ rCBF during viewing of disorder-related stimuli or neutral stimuli. Beato-Fernandez et al. [34] examined rCBF across AN-Restricting (AN-R), AN-Binge-Purge (AN-BP), BN-Non-Purging (BN-NP), BN-Purging (BN-P) and HCs using three SPECT imaging procedures during rest, calm visual stimulus, and after seeing their own body image photographs. In the BN-P and AN-R groups, increased rCBF in the right temporal area was found when going from neutral to body image visual stimulus (Beato-Fernandez et al., [34]). Karhunen and colleagues [63] also found increased rCBF during exposure to food images compared to neutral images, in the left frontal and prefrontal cortices in obese BED participants compared to obese non-BED and HC participants. Nozoe and colleagues [64] completed SPECT before and after AN, BN and HC women ate a slice of cake and found the BN group had the highest rCBF before eating in the left temporal and bilateral inferior frontal regions, but the lowest cortical activity after eating. Finally, Delvenne et al. [65] completed a resting-state PET with a chemical marker to assess cerebral glucose metabolism, finding women with BN demonstrated absolute glucose hypometabolism globally and regionally, most notably in the parietal and superior frontal cortices, compared to HCs.

## Discussion

This systematic review identified and reviewed 32 papers investigating neural differences between individuals with BN and / or BED and HC groups [18, 22–26, 34–39, 42–47, 49–59, 63–65]. Included in this review were sixteen studies on participants with BN, eleven studies on participants with BN and AN, three studies on participants with BN and BED and two studies on participants with BED. The objectives of this review were first, to provide a synthesis of published studies on neuroimaging in BN and BED. Due to the heterogeneity of the 32 studies, results were reviewed according to the method of the study and type of neurological test completed (MRI, fMRI, SPECT and/or PET). The evaluation of literature indicates that it is too early to make any definite conclusions about the neuroimaging profile of individuals with BN or BED. The diverse range of neuroimaging procedures and stimuli used during testing coupled with small sample sizes impeded the ability to draw many clear conclusions. Despite this, a discussion is presented below which attempts to highlight the findings in a meaningful manner.

In relation to the second objective, to identify neurobiological studies that will assist in elucidating the apparent decreasing clinical utility of OBEs and SBEs, no studies were found wherein comparisons were made between participants reporting OBEs versus SBEs. It is worth noting that a number of studies in this review compared sub-groups of participants based on *severity* [24, 26, 35, 39, 45, 47, 52, 58], which was exclusively defined as the *frequency* of symptoms, rather than an attempt to quantify the actual amount of food consumed during a BE. The frequency of BE and purging in BN is considered an important predictor of therapeutic outcome [26] and this review certainly highlights that more aberrant neurobiological activity is reported in patients with more frequent binge eating or bulimic episodes.

If it is accepted that the severity of bulimic symptoms in the context of clinical assessment and neuroimaging research is based on the frequency of binge and / or purge episodes, it raises further doubt regarding the clinical utility and validity of continuing to attempt to quantify individuals’ binges as OBEs or SBEs.

### MRI studies

Seven studies included in this review reported on data obtained from MRI [22, 24, 35–39]. The findings of MRI studies were mixed; most identified structural changes in the brains of BN and BED patients. Of the seven studies, only one included both BN and BED participants [24]; two studies recruited BN and AN participants [36, 37]; and four investigated BN participants only [22, 35, 38, 39]. Of the two studies investigating BN and AN participants, one reported similarities in both groups in terms of smaller pituitary glands, which the authors postulated may have atrophied following prolonged malnutrition [37]. The pituitary gland is central in the hypothalamic-pituitary-gonadal axis and is critical for normal sexual and hormonal development in puberty. A recent study [66] found age, puberty stage, testosterone and estradiol levels predicts pituitary volume in adolescents, which may be relevant to this finding considering the onset of illness is usually during adolescence. The second study reported the midbrain and thalamus demonstrated significant volume reduction only in the AN group, not in the BN and HC groups [36]. The thalamus is thought of as the ‘gate keeper’ to the cerebral cortex and is centrally involved in taste and gustatory processing, before communicating sensory information to the frontal region and insula for further processing [67].

One MRI study compared BN, BED and HC groups [24], finding evidence of structural changes in the reward-learning circuit; specifically, increased grey matter volumes in the medial orbitofrontal cortex (OFC) in both BN and BED. This finding may reflect differential reward processing as the OFC is implicated in the hedonic value of food stimuli [24, 61]. fMRI studies in this systematic review have also highlighted alterations in the medial OFC associated with processing visual food reward cues in eating disorder groups compared to HCs [52, 54, 63]. Schafer and colleagues [24] also found increased volume in the VS in BN, which was significantly and positively correlated with a lower BMI and increased frequency of purging. The central role of the striatum, in particular the VS, in the processes of decision making, reward and motivation, is now well-accepted based on a large number of human neuroimaging studies [68]. This finding, in combination with increased medial OFC volume, suggests a joint alteration of food reward processing and instrumental behaviour to reduce the chance of weight gain as the end goal [24].

The four remaining MRI studies compared BN and HC groups only. Three studies [22, 35, 38] reported structural changes in terms of cortical atrophy or volume reduction in the BN group, firstly in the ratio of cerebral to cranial area [35] and in the second paper, in the inferior frontal grey matter [38]. We believe that Hoffman and colleagues [35] made the first critical finding of a significant positive correlation between illness severity (binge frequency) and structural changes in the brain. The third paper to identify structural changes found volume reduction in the caudate nucleus within the frontostriatal circuit [22]. Lastly, Galusca et al. [39] reported widespread impairment in serotonergic activity in BN patients during PET and MRI, which is consistent with well-known research that serotonergic activity is disturbed in BN, and serotonin-modulating medications decrease BN symptoms independently of their anti-depressant effects [69]. It is notable that Galusca and colleagues’ [39] study recruited only *severe* BN-P participants, where the frequency of binge-purge episodes had to be a minimum of at least once daily for a minimum of six months, however we consider this group to be more representative of individuals likely to seek treatment, due to the distress associated with symptoms at this level.

The collective MRI results reveal several key findings. Firstly, in BN groups structural changes have been consistently reported, firstly in the ratio of cerebral to cranial area volume [35]; decreased inferior frontal grey matter [38]; reduction within the frontostriatal circuit, specifically in the caudate nucleus [22]; and volume reduction in the pituitary gland [37]. In one study [24], increased medial OFC volumes characterised BN and BED patients while increased volumes within the VS, dorsal striatum, medial and lateral OFC was found in BN patients only, and BED patients had increased volume within the ACC. The only combined PET and MRI study included in this review highlighted widespread serotonin dysfunction in individuals with severe BN [39].

### fMRI studies

Twenty-one studies in the present review used fMRI as the primary method of investigation [18, 23, 25, 26, 41–57]. Studies included in this review using fMRI techniques yielded a broad range of results. With regards to studies of food-specific stimuli, results suggested that patients with bulimic pathology have less activation in gustatory and reward regions before and during eating, which may mediate the tendency to over-consume and binge eat [58]. Schienle and colleagues [52] reported greater activation in the insula and the ACC in BN patients, which was positively associated with frequency of BEs. An interesting finding in this study was the differential response in BED patients, producing enhanced reward sensitivity and medial OFC responses compared to BN and HC groups [52]. In response to a measure of inhibitory control (a food-specific no-go task), BN participants demonstrated decreased activation in the right postcentral and precentral gyrus, and right dorsal striatum (caudate nucleus, putamen); diminished frontostriatal brain activation was associated with high symptom severity in BN [26]. This is of note as diminished frontrostriatal brain activation may contribute to symptom severity. Uher and colleagues [54] also found diminished activity, however this was in the anterior and lateral PFC of BN patients, in response to food images. The lateral PFC is associated with suppressing unwanted behaviours, hence impaired activity in this region may underlie the disinhibition of eating behavior [54, 70]. Aberrant responding in the PFC is common to eating disorders, obsessive-compulsive disorders (OCD) and addictive disorders and may underlie some of the compulsive features of these illnesses, while the OFC and ACC are areas where the convergence of processing of emotional information and are consistently implicated in OCD and affective disorders [54, 71, 72].

In response to food images used as interference for behavioural performance in the Stroop task [33], women with BED had stronger activation in the VS, while women with BN had greater activation in the premotor cortex and dorsal striatum [44]. The aberrant activity of the ventral and dorsal frontostriatal regions in response to food images may underlie increased reward sensitivity and habitual binge eating behaviours in BED and BN. In another study, BN patients had greater activation in areas associated with somatosensory and motor responses in the right insula cortex and post-central gyrus when asked to think about eating the food shown in photographs [42].

Six fMRI studies included in this review utilised body image-specific stimuli [49, 50, 53, 55, 56, 59] and results were mixed. One study [55] reported the activity of the lateral fusiform gyrus and parietal cortex were decreased in eating disorder participants [55] while in another study, in response to morphed (‘fat’) photographs of their own and another woman’s bodies, BN participants did not show significant activation of the amygdala or PFC, unlike AN and HC groups, however they did demonstrate activation in the occipital and parietal lobes [59]. This finding was partly in contrast to Mohr and colleagues [50], who found women with BN did not demonstrate a modulation in the occipital cortex while looking at distorted photographs of their own bodies, unlike HC women. Also, the BN group failed to execute the middle frontal gyrus (MFG) during a body size estimation task, while the HC group did, suggesting a reduced spatial manipulation capacity in BN participants [50]. The MFG is believed to be the gateway between top-down and bottom-up attentional processes [73]. In response to negative body image words, the BN and HC groups differed from the AN group by not demonstrating significant activation of the right amygdala, while the BN and AN-BP groups demonstrated left mPFC activation [49]. Spangler and colleagues [53] also found increased mPFC activity in BN, specifically while looking at ‘fat’ computer generated images of female bodies compared to HCs, with peak activity in the pregenual anterior cingulate cortex (pgACC), which is thought to be the affective area of the ACC, and has a key role in emotional processing and emotion regulation possibly through modulation of the amygdala [53, 74]. Also reporting differential activity in the amygdala, Vocks and colleagues [56] found women with BN and HCs had decreased amygdala activity relative to women with AN when looking at photographs of another woman wearing a bikini, however when viewing photographs of their bodies while wearing a bikini, AN and BN groups had reduced inferior parietal lobe activity. The involvement of the amygdala in processing body-related stimuli underlines the dominant emotional processing taking place in AN, while the available data suggest that in BN, processing involves more spatial perception and comparisons in size [49] with the lateral occipital cortex being a key region for the perception of the human body [75]. Involvement of the mPFC may reflect attempted emotional regulation in response to this disorder-specific stimuli [49].

A further six fMRI papers investigated the neural correlates of decision making or learning paradigms [18, 23, 25, 43, 45, 46]. Decreased bilateral VS activity during anticipatory reward and loss processing was reported in BED participants compared to obese participants (without BED), alongside diminished PFC and insula activity during outcome processing [23]. Abnormal functioning of the anterior hippocampus and frontostriatal regions were reported in adolescent girls with BN, suggesting that imbalances in neural circuits of control and reward may develop earlier in the course of the illness [43]. Two studies using spatial incompatibility tasks [45, 46] both reported hypoactivity within the frontostriatal circuits of women and female adolescents with BN, which again highlights impaired self-regulatory processing. During a measure of probabilistic category learning, women with BN demonstrated diminished activity in the areas of the brain associated with episodic memory during early learning, and late increased activity in areas associated with category learning [25]. These results indicate women with BN may have suppressed early learning, or fast and flexible learning, which may translate into short-term memory deficits. Lastly, Seitz and colleagues [18] demonstrated significantly altered neural activity in all three attentional networks (alerting, reorienting and executive attention) in women with BN.

The three remaining fMRI studies included in this review didn’t fall into any of the previous categories, hence are discussed here [47, 51, 57]. Firstly, when processing negative self-referent emotive words related to eating disorders BN patients demonstrated hypoactivation in parietal, occipital and limbic regions including the amygdala [51]. Reduced activation of the amygdala highlights deficits in emotional appraisal and processing in BN patients, while the hypoactivation of the parietal and occipital lobes indicates down-regulating of attention and visual processing respectively, hence the authors proposed the BN group may have avoided engaging in the task due to the aversive valence of the stimuli. In the only study to complete a resting-state fMRI, results showed hyperconnectivity of the cerebellar network, heavily involved in processing emotion, and the parietal cortex, as well as increased bilateral connectivity of the cerebellar intrinsic connectivity network with the temporal poles, which are thought to have a role in processing social behavior and emotional stimuli in BN and AN groups [57]. In the BN group only, increased connectivity of the cerebellar network with the ACC was reported [57]. The differential activation of the ACC in BN emerged as a common finding within other papers in this review [45, 52].

In the third study, significant volume reduction in the frontal and temperoparietal regions in adult and adolescent BN groups was reported and furthermore, an inverse correlation whereby greater reduction in inferior frontal regions was found in patients with higher symptom severity, age and Stroop interference scores [47]. Volume reduction abnormalities, specifically on the surface of the inferior frontal lobe, may reflect a global change in this region and possibly contribute to deficits of self-regulation in feeding and other behaviours seen in BN [47].

The collective fMRI findings highlight several key findings. Hypoactivity in the frontostriatal circuits was reported in four fMRI studies of BN patients in the acute illness state [26, 43, 45, 46], which may promote the impulsive and dysregulated eating and emotion-related behaviours seen in this illness. It should be noted that one study reported hyperactivity in the frontostriatal circuits [25]. We suggest that both of these profiles of activity - hypo or hyperactivity – indicate an inefficiency of this system in BN. Next, following on from Hoffman et al’s [35] early MRI finding of a significant positive correlation between the degree of cortical atrophy and illness severity / binge frequency in BN, two additional fMRI studies have also highlighted an important link between illness severity in BN and aberrant neurological activity. This includes increased insula activation [52], involved in the neurological processing of food-related stimuli [42], and diminished frontostriatal activity [26], which contributes broadly to emotion, motivation, movement processes and importantly, is thought to underlie self-regulatory control. Two studies reported diminished ‘fast and flexible’ (early) learning [25] and attentional capacity [18] in BN participants.

### SPECT and PET studies

Three studies in this review used SPECT imaging; two studies investigating participants with BN and AN, and one study investigating participants with BED. All three studies found increased rCBF in response to disorder-related stimuli, in the right temporal area in BN-P [34], left frontal and PFC in BED [63] and left temporal and bilateral inferior frontal regions in BN participants [64]. Increased blood flow to the right temporal lobe has been associated with phobic responses [76] and the results of Beato-Fernandez and colleagues’ [34] were proposed as a discriminant variable, supporting the idea that the BN sub-phenotypes (BN-P and BN-NP) are significantly different in relation to neural responses and psychopathology. In obese BED patients, increased rCBF was found in the left frontal and PFC while looking at a portion of real food after fasting overnight [63]. Previous research has found obese non-BED women had increased rCBF in the opposite (right) hemisphere during food exposure [77], which is in line with previous evidence of cerebral asymmetry in eating disorders across diagnostic groups [78, 79]. Increased rCBF in the left temporal region and bilateral inferior frontal regions was reported in women with BN before eating a slice of cake, however this group had the least post-eating activity compared to AN and HC groups [64], highlighting that BN and AN groups demonstrated an opposite pattern of frontal activity to the slice of cake. Finally, one study used resting-state PET, finding global and regional glucose hypometabolism in BN participants, with significantly reduced metabolism in the parietal cortex [65]. Phillipou et al [80] reported the same finding in their systematic review of the neurobiology of AN patients, which is not surprising given the parietal cortex is implicated in body image perception [81].

### Limitations

The primary limitation of this systematic review is the heterogeneity of the methodologies across the included studies, with regards to the neuroimaging test performed and the stimuli or paradigms used during the tests, in combination with small sample sizes. Furthermore, these factors precluded a meta-analysis from being conducted. Although the inconsistent methodologies and small sample sizes decrease the rigour and the degree to which we can interpret these results collectively, we nonetheless believe our review will contribute to increased understanding of the neurobiological mechanisms underlying binge eating in BN and BED. Following on from Van den Eynde and colleagues [21], the findings were discussed collectively wherever possible because of the dimensional approach we also took to binge eating, however it is not clear whether BN and BED share more neurological features than not, as only three of the thirty-two studies in this review compared BN and BED groups.

Neurological performance is at least partly state-dependent, yet hunger/satiety, or the time of the last meal prior to participation were not controlled in the majority of studies. It is therefore impossible to delineate the contribution of potential caloric deprivation, which can lead to metabolic and neuroendocrine changes in the brain [25]. Given hunger may result in heightened neural reward activation in response to eating a portion of food, differences between people with and without BN or BED may be more evident after eating [58]. Furthermore, due to the known cycles of actual or attempted restriction following binge episodes, giving a snack at a prescribed time prior to testing may have leveled differences in reward activity.

Similarly, no studies in this review controlled for stage of menstrual cycle which may have affected results; there is evidence of neurofunctional modulation of the reward system by gonadal steroid hormones while ovarian steroids modulate reward-evoked neural activity in humans [82]. Completing an fMRI study, Dreher and colleagues [82] found that during the midfollicular phase (days 4-8 post-onset of menses) women anticipating uncertain rewards activate the orbitofrontal cortex and amygdala more than during the luteal phase (6-10 days after lutenising hormone surge). Also, we believe only one study controlled for stage of treatment by completing the neurological scan before, or in the first week of, cognitive therapy [64]; most did not account for this potentially confounding variable. With regards to medication, there was heterogeneity in terms of whether psychotropic medication, SSRI’s or antidepressants were an exclusion for participation. One study addressed the potential confounding factor of SSRI medication on neurological activity and performance, by completing a second analysis of their fMRI data excluding participants taking medication, finding that the two groups differed in neural activity [54].

With regards to additional participant characteristics, men were not included with the exception of one, or possibly two studies [23, 34]. Also, the majority of studies recruited clinical samples of eating disorder patients versus community-based recruitment yet particular psychiatric comorbidities were excluded in some studies, for example depression. Approximately 90% of people with BN will have a lifetime diagnosis of another psychiatric disorder [83], while for those with BED, the greatest lifetime comorbidity is for other eating disorders followed by depression and bipolar disorder [28]. It is clear that for individuals with clinical severity binge eating, psychiatric comorbidities are the rule rather than the exception, hence one must question the decision to exclude participants with psychiatric comorbidities given they are representative of the norm.

### Recommendations for future research

Considering poor treatment outcomes for many individuals with BN and BED, evidence-based treatment requires critical further development and is very unlikely to come from a refinement of traditional psychological treatment, or ‘talking therapy’, alone [10]. Talking therapies target ‘top-down’ cognitive processes; however, it is apparent that developing greater insight into the neurological mechanisms of BN and BED, in particular behavioural inhibition deficits, which are the hallmark of BN and BED, will inform brain-based treatment frameworks [26]. Repetitive transcranial magnetic stimulation (rTMS), a form of neuromodulation, has been shown to decrease cue-induced food cravings for individuals with bulimic-type eating disorders [17]. A second potential treatment approach warranting further research is the use of neurofeedback in the treatment of BN or BED, which is a form of biofeedback that trains individuals to actively and voluntarily regulate their neural activity in response to real-time feedback via a brain-computer interface [84]. The value of neurofeedback is that, unlike fMRI, it provides a way of assessing neural activity and responses via its effect on behavior and as such, can be seen as ‘bridging the gap’ between traditional psychological treatment approaches and our current neurobiological knowledge of BN and BED [84]. Electroencephalography (EEG) feedback has been extensively investigated in ADHD and overall, findings suggest its use in this group translates into significant and lasting improvements in cognition and behavior that are equal to or better than medication [84, 85]. Considering the overlap between BN, obesity and ADHD [20, 86], combined with the results discussed in this review highlighting deficits of frontostriatal and attentional functioning, this suggests EEG neurofeedback certainly presents potential for the treatment of BN and BED.

While the cost of conducting neurobiological research is extremely high relative to other research, future research design utilizing larger sample sizes and power would contribute much more knowledge to this area, thereby enhancing the rigor that this area of research warrants. Additionally, with increased awareness of the need for rigorous study design and the lack of data reproducibility, especially in neuroimaging research, future studies would be well-placed to design and register pre-clinical experiments and plan carefully around how neuroimaging data is collected and analysed in advance.

This review only included studies with patients currently in the ill-state of either BN or BED, so it is not possible to speculate on whether the differences reported were a precipitant or consequence of the mental illness. It is accepted that eating disorders demonstrate considerable pathoplasticity [10]. Therefore, larger collaborative studies conducting neurobiological testing at numerous time points with the same cohort of BN and BED patients would help to elucidate this question and present more robust research design. Similar studies have been completed in AN which have provided critical information regarding the neurological changes that take place in the acute ill phase of the illness and furthermore, how the brain ‘recovers’ following refeeding [87].

In this review, a relationship between symptom severity and neural activity was established [35, 42, 52]. Additional research is required to determine whether these differences in neural activity based on clinical severity could provide a predictor of treatment response. Finally, only one [23], or possibly two [34], studies of the thirty-two papers in this review included male BN or BED patients. There is a clear need to include male patients in future neuroimaging studies of BN and BED.

## Conclusions

This review demonstrates a relationship between symptom severity and aberrant neural activity, and deficits in self-regulatory neural processes related to decreased activity in the frontostriatal circuits in patients with BN or BED. The extent to which the structural and functional changes described in this review occur as a result of the eating disorder, or contribute to its development remain to be established.

## Abbreviations

5-HT: Serotonin
ACC: Anterior cingulate cortex
AN: Anorexia nervosa
AN-BP: Anorexia nervosa-binge purge type
AN-R: Anorexia nervosa-restricting type
BE: Binge episode
BED: Binge eating disorder
BMI: Body mass index
BN: Bulimia nervosa
BN-P: Bulimia nervosa-purge type
CN: Caudate nucleus
DA: Dopamine
DLC: Dorsolateral cortex
Dstr: Dorsal striatum
fMRI: Functional magnetic resonance imaging
GMV: Grey matter volume
HC: Healthy control participants
Insula: Insular cortex
LOC: Loss of control
MFG: Middle frontal gyrus
mPFC: Medial prefrontal cortex
MRI: Magnetic resonance imaging
OBE: Objective binge episode
OFC: Orbitofrontal cortex
OSFED: Other Specified Feeding and Eating Disorder
PET: Positron Emission Technology
PFC: Prefrontal cortex
SBE: subjective binge episode
SD: Standard deviation
SPECT: Single-Photon Emission Computed Tomography
Th: Thalamus
VS: Ventral striatum

## Footnotes

1 Binge episodes (BEs) in this paper refer exclusively to binge eating episodes involving the consumption of food, excluding any other substance or item in the binge episode.
